# Atorvastatin Shows Limited Disease-Modifying Effects in an A53T α-Synuclein Mouse Model of Parkinson’s Disease

**DOI:** 10.1007/s12640-026-00825-y

**Published:** 2026-09-09

**Authors:** Lijian Wei, Junkai Hua, Shuangfeng Tang, Jianming Lei, Guozhi Li, LiWei Wei, MingShu Mo

**Affiliations:** 1 Department of Geriatrics, Maoming People’s Hospital, Maoming, Guangdong China; 2 Guangzhou Medical University, Guangzhou, Guangdong China; 3 Department of Oncology I, Maoming People’s Hospital, Maoming, Guangdong China; 4 Department of Emergency, Guangdong Provincial Farm Reclamation Central Hospital, Zhanjiang, Guangdong China; 5 Department of Neurology, The First Affiliated Hospital of Guangzhou Medical University, Guangzhou, Guangdong China

**Keywords:** Parkinson’s disease, α-Synuclein, Atorvastatin, Molecular docking, Transcriptomics

## Abstract

Neuroprotective effects of statins in Parkinson’s disease (PD) remain uncertain, and their activity in genetic α-synuclein (αSyn) disease models has been insufficiently characterized. We evaluated atorvastatin (ATO) in a mouse model with nigral overexpression of human A53T-mutant αSyn. Mice received ATO by oral gavage at 10 mg/kg/day for 5 weeks and were assessed using behavioral testing, neuropathological assessment, brain transcriptomics, and molecular docking. ATO inhibited cholesterol biosynthesis-related transcriptional programs and broadly remodeled lipid metabolism-associated networks, but did not lead to functional or histopathological benefit. ATO did not ameliorate motor deficits, restore dopaminergic markers, or reduce αSyn protein levels or pSer129-αSyn immunoreactivity. Transcriptomic analysis further showed that ATO failed to reverse the core disease-associated signature induced by A53T αSyn overexpression and instead increased *SNCA* mRNA. Targeted RNA-seq and western blot analyses showed no parallel increase in *Prkn*,* Gba1*, or *Lamp2* transcript abundance or in PARKIN, GBA1, or LAMP2A protein expression. Molecular docking, used here as an exploratory structural comparison, suggested a relatively weak predicted interaction between ATO and αSyn when compared with several other statins and provided supportive context for the lack of efficacy. Overall, our findings indicate limited efficacy of ATO in this αSyn-driven setting and support further comparative evaluation of individual statins across complementary PD models.

**Graphical Abstract:**

**Figure:**
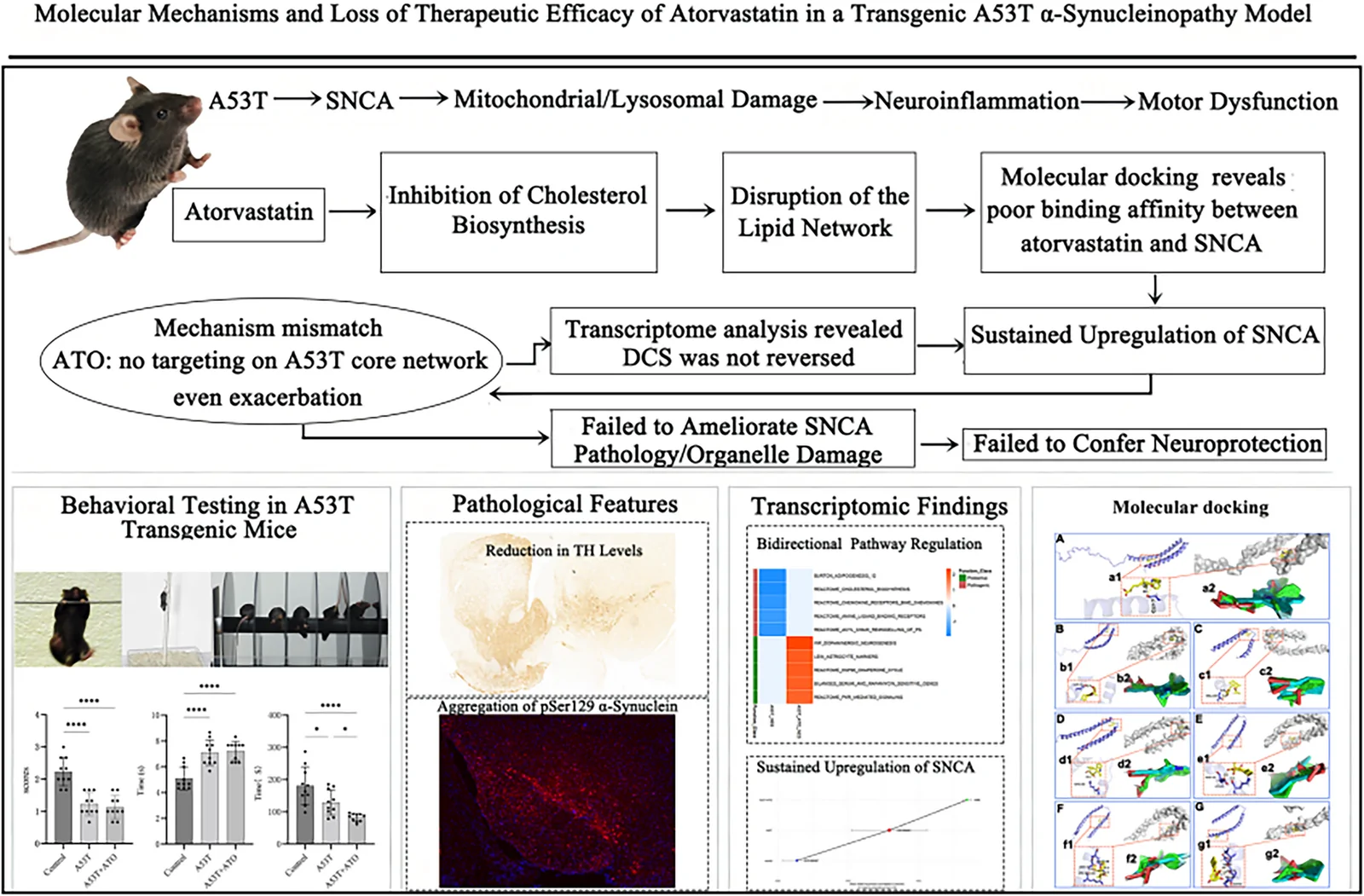

**Supplementary Information:**

The online version contains supplementary material available at https://doi.org/10.1007/s12640-026-00825-y.

## Introduction

Parkinson’s disease is characterized by the abnormal accumulation of αSyn and the progressive degeneration of dopaminergic neurons (Calabresi et al. 2023; Kim et al. 2023). Among the many processes linked to PD, the αSyn pathway is of primary importance, as confirmed by consistent genetic studies (Bellini et al. 2025; Lodge and Agin-Liebes 2026; Mohammadi et al. 2025). In familial PD, coding synuclein alpha *(SNCA)* mutations such as A53T and whole-gene multiplications increase αSyn burden and accelerate disease onset (Oliveira et al. 2015; Ye et al. 2023). In sporadic PD, the mechanism is less direct but leads to a similar conclusion. Risk variants near *SNCA* may contribute to PD risk through regulatory mechanisms rather than coding sequence change (Prahl et al. 2023). Recent single-nucleus multi-omics studies further suggest that these regulatory effects can reshape transcriptional programs in vulnerable neuronal populations (Shwab et al. 2024). Taken together, these observations support the view that, in at least a substantial subset of PD, αSyn is not simply one pathological feature among many, but part of the central disease process. Even so, treatments that can meaningfully modify αSyn-driven pathology remain elusive.

Statins have been proposed as candidates for disease modification in PD because, beyond lowering cholesterol, they also exert anti-inflammatory and antioxidant effects and may influence neurobiological pathways relevant to disease progression (Jeong and Lee 2025). However, preclinical and clinical findings remain inconsistent. Protective effects are frequently reported in toxin-based models such as 1-Methyl-4-phenyl-1,2,3,6-tetrahydropyridine (MPTP) (Du and Bu 2021; Ghosh et al. 2009; Marques et al. 2018; Yan et al. 2020a, b), whereas evidence from genetic models that more directly recapitulate chronic proteinopathy is limited and less conclusive. Lovastatin has been reported to reduce αSyn-associated pathology in a transgenic model (Koob et al. 2010), but broader studies of mevalonate-pathway modulation in PD remain heterogeneous and mechanistically underdeveloped (Saeedi Saravi et al. 2017). In clinical observational studies, the association between statin use and PD is unclear (Jeong et al. 2021). These discrepancies raise an important question: when a statin is administered in a genetic αSyn-overexpression model, does it modify the core disease network or merely induce parallel molecular changes without functional benefit?

Among commonly used statins, ATO is a particularly relevant compound to test because it has shown anti-inflammatory and neuroprotective effects in toxin-based PD paradigms, yet its activity in genetic α-synucleinopathy models remains poorly defined. Here, we evaluated ATO in an A53T αSyn overexpression mouse model using behavioral testing, neuropathological assessment, brain transcriptomics, and exploratory molecular docking. We specifically asked whether ATO could alleviate motor and nigrostriatal pathology, whether it could alter the core disease-associated transcriptional program, and whether exploratory structural comparison might help interpret its activity in an αSyn-driven setting. This design allowed us to assess efficacy while also examining possible reasons why clear pathway-level activity might fail to translate into measurable neuroprotection.

## Materials and Methods

### Experimental Animals

Male wild-type C57BL/6J mice (6–8 weeks old, weighing 20–25 g) were purchased from Guangdong Pharmapack Biotech Co., Ltd. All animals were housed under specific pathogen-free (SPF) conditions in a controlled environment (temperature: 22 ± 2 °C, humidity: 60 ± 5%, 12-hour light/dark cycle) with ad libitum access to food and water. All experimental procedures were approved by the Experimental Animal Ethics Committee of Guangzhou Medical University (Reference: 2023028) and strictly adhered to the National Institutes of Health (NIH) Guide for the Care and Use of Laboratory Animals. Mice were randomly assigned to three groups: a wild-type group injected with the corresponding empty rAAV9 vector (Control group; *n* = 12), a wild-type group injected with rAAV9-A53T (A53T group; *n* = 12), and a wild-type group injected with rAAV9-A53T and treated with ATO (A53T + ATO group; *n* = 12). The empty rAAV9 vector and rAAV9-A53T vector were diluted in sterile PBS using the same procedure and administered using the same stereotaxic injection protocol. A separate ATO-only control group was not included because pilot experiments showed that ATO did not alter tyrosine hydroxylase (TH) expression in wild-type mice, consistent with previous reports (Kumar et al. 2012; Marques et al. 2018). On this basis, an additional ATO-only control group group was not pursued, in order to limit animal use in accordance with the 3R (Replacement, Reduction, Refinement) principles (Kirk 2018; Sneddon et al. 2017; Verderio et al. 2023) while preserving the main disease-versus-treatment comparison. Behavioral testing was performed longitudinally before tissue collection. During the experimental period, two mice in the A53T + ATO group died and were therefore unavailable for subsequent analyses. To maintain balanced group sizes across downstream analyses, two mice from each of the Control and A53T groups were randomly selected and not included in the final analysis set. The final analyzed sample size was therefore 10 mice per group. These 10 animals per group were used for behavioral analysis and subsequently entered terminal tissue collection and assay allocation as described below.

### Stereotaxic Injection and Viral Transduction

The recombinant adeno-associated virus serotype 9 carrying human A53T mutant αSyn (rAAV9-A53T) and the corresponding empty rAAV9 vector was obtained from Shandong Weizhen Biotechnology Co., Ltd. (titer: 1.0 × 10¹³ vector genomes/mL). The virus was diluted in sterile phosphate-buffered saline (PBS, pH 7.4). Mice were anesthetized with an intraperitoneal injection of 0.6% sodium pentobarbital and placed in a stereotaxic frame. Coordinates for the substantia nigra (SN) pars compacta were determined relative to bregma: anteroposterior (AP) −3.0 mm, mediolateral (ML) ± 1.3 mm, dorsoventral (DV) −4.75 mm. A total volume of 0.5 µL virus suspension was injected into each hemisphere at a rate of 0.1 µL/min. The needle was left in place for 5 min before slow withdrawal. Mice in the A53T and A53T + ATO groups received bilateral intranigral injections of rAAV9-A53T, whereas mice in the Control group received bilateral injections of the corresponding empty rAAV9 vector using the same coordinates, injection volume, injection rate, and postoperative procedure.

### Drug Administration and Tissue Collection

Starting on the day after stereotaxic surgery, mice in the ATO treatment group received daily oral gavage of ATO (10 mg/kg dissolved in saline) for 5 consecutive weeks, while the Control and A53T groups received an equal volume of saline. The daily dose of 10 mg/kg was selected based on a previous MPTP mouse study that used the same daily dose and reported changes in motor performance, TH expression, pSer129-αSyn, oxidative stress, and autophagy-related measures (Yan et al. 2020a). At the beginning of week 6, mice were deeply anesthetized and processed for tissue collection based on their pre-determined endpoint allocation. Within each group, 6 mice were allocated to molecular and ultrastructural analyses, while the remaining 4 mice were reserved for histological studies. For the 6 mice assigned to molecular analyses, brains were removed and hemisected sagittally immediately after collection. One hemisphere from each animal was randomly designated for western blotting. From the opposite side, tissue was further distributed according to the downstream assay: the SN from 2 mice per group was processed for transmission electron microscopy (TEM), whereas the entire hemisphere from 3 mice per group was used for RNA sequencing. In all 6 mice, the contralateral SN was collected for western blotting, while the remaining hemispheres were reserved as backup tissues. This arrangement allowed protein-level readouts to be obtained consistently across the full molecular cohort, while still preserving tissue for transcriptomic and ultrastructural analyses. Because the SN is a small and anatomically compact structure, dissection was performed on a chilled plate immediately after brain removal to reduce tissue degradation. Using a rodent brain matrix and the mouse brain atlas as anatomical guidance, a coronal cut was made just rostral to the SN to isolate the ventral midbrain block. The SN was then carefully microdissected under direct visual guidance with fine forceps and a surgical blade, using the cerebral peduncles laterally and the interpeduncular fossa medially as landmarks. Depending on the assigned endpoint, dissected tissue was either snap-frozen in liquid nitrogen and stored at − 80 °C for subsequent molecular analysis or immediately immersed in ice-cold fixative for TEM processing. The remaining 4 mice in each group were used for histological analyses. These animals were transcardially perfused with ice-cold PBS followed by 4% paraformaldehyde (PFA). Their brains were then removed, post-fixed in 4% PFA, and prepared for cryostat sectioning for immunohistochemistry and immunofluorescence.

### Western Blotting

SN tissues were homogenized in Radioimmunoprecipitation Assay (RIPA) lysis buffer containing protease and phosphatase inhibitors. After centrifugation, the supernatant was collected, and protein concentration in the supernatant was measured using the bicinchoninic acid assay (BCA). Equal amounts of protein (typically 20 µg) were subjected to sodium dodecyl sulfate-polyacrylamide gel electrophoresis (SDS-PAGE) and then transferred onto polyvinylidene fluoride (PVDF) membranes. After transfer, membranes were washed with Tris-Buffered Saline with Tween-20 (TBST) and blocked in 5% non-fat milk in TBST for 1 h at room temperature. The membrane was subsequently divided according to the molecular masses of the target proteins and loading controls, so that each portion could be incubated with the appropriate primary antibody. Incubation with primary antibodies was carried out overnight at 4 °C. The study utilized the following primary antibodies: α-synuclein (1:1000, ab138501, Abcam), TH (1:1000, 25859-1-AP, Proteintech), PARKIN (1:1000, RM5042, BioDragon), GBA1 (1:1000, 2055, Genuin Biotech), LAMP2A (1:1000, RM0727, BioDragon), β-actin (1:5000, AF7018, Affinity Biosciences), and GAPDH (1:5000, 81640-5-RR, Proteintech). After washing, membranes were incubated for 2 h at room temperature with horseradish peroxidase-conjugated secondary antibodies (goat anti-mouse, 1:5000, 62–6520, Invitrogen; goat anti-rabbit, 1:5000, 32460, Invitrogen). Signals were developed using Super ECL Detection Reagent (36208ES, Yeasen) and captured with a chemiluminescence imaging system (Syngene, Cambridge, UK). Densitometric analysis was performed using ImageJ software (version 1.54, National Institutes of Health, USA; https://imagej.nih.gov/ij/). To ensure accurate quantification, the intensity of each target protein band was normalized to the corresponding β-actin or GAPDH signal in the same lane, according to the loading control used for each blot. This experiment included six independent biological replicates, and the combined data were analyzed using rigorous statistical methods.

### Immunofluorescence and Immunohistochemistry

Mice were transcardially perfused with ice-cold PBS followed by 4% PFA for tissue immunofluorescence and immunohistochemical staining. Brains were post-fixed in 4% PFA for 24 h, dehydrated in a sucrose gradient (10%, 20%, 30%) for 48 h, embedded in Optimal Cutting Temperature compound, and coronally sectioned at 16 μm thickness on a cryostat. To ensure consistent sectioning locations, the mouse brain atlas (Paxinos and Franklin, 4th edition) was consulted, and the symmetry of the corpus callosum and striatum served as anatomical landmarks during embedding. The cryostat blade was adjusted to be perpendicular to the midline. Sections were collected continuously from the level of bregma to the caudal extent of the SN. The coronal plane with the largest SN area (3.16 mm posterior to bregma) was chosen, and 12 sections were collected, with 6 designated for immunohistochemistry and 6 for immunofluorescence.

For immunofluorescence staining, sections were air-dried at room temperature for 30 min, rehydrated in PBS for 10 min, and underwent antigen retrieval in preheated citrate buffer (pH 6.0) at 95 °C for 20 min. Following cooling to room temperature, sections were permeabilized with 0.3% Triton X-100 in PBS (Beyotime, Shanghai, China) for 15 min, then incubated with goat serum (AR0009, Boster, China) at room temperature for 2 h to block nonspecific binding sites.

After blocking, sections were incubated with the corresponding primary antibodies overnight at 4 °C. The primary antibodies used included: rabbit anti-phosphorylated α-synuclein (Ser129) antibody (1:500, ab51253, Abcam, UK), rabbit anti-IBA-1 antibody (1:500, 019–19741, Fujifilm Wako, Japan). Following PBS washes, sections were incubated with fluorescent secondary antibodies for 1 h at room temperature in the dark. The secondary antibodies used were: Alexa Fluor^®^ 555-conjugated goat anti-rabbit IgG (1:1000, 4413, Cell Signaling Technology, USA) and Alexa Fluor^®^ 488-conjugated goat anti-mouse IgG (1:1000, 4408, Cell Signaling Technology, USA). Cell nuclei were counterstained with a DAPI-containing mounting medium (F6057, Sigma-Aldrich, Merck, Germany). All fluorescent images were acquired using a Leica DM6 upright fluorescence microscope (Leica Microsystems GmbH, Wetzlar, Germany) and subsequently analyzed.

To perform immunohistochemical analysis, frozen tissue sections underwent antigen retrieval by heating in sodium citrate buffer (pH 6.0) using a microwave. Following three washes with PBS, the sections were treated with 0.3% Triton X-100 for 15 min to enhance permeability. Endogenous peroxidase activity was then quenched by incubation with 3% hydrogen peroxide (Wuhan Boster Biological Technology Co., Ltd.) for 15 min at room temperature. Non-specific binding was reduced by blocking the sections with goat serum (AR0009, Boster, China) at 37 °C for 60 min. The sections were subsequently incubated with rabbit anti-tyrosine hydroxylase antibody (1:500, 25859-1-AP, Proteintech, China) overnight at 4 °C. After washing three times with PBS, a biotin-conjugated secondary antibody (abs996, Absin, Shanghai) was applied for 60 min at room temperature. Immunodetection was carried out using a commercial immunohistochemical staining kit (Shanghai Gene Technology Co., Ltd.). Finally, the sections were dehydrated through a graded ethanol series (75%, 90%, and 100%), cleared in xylene substitute (Guangxi Cenxi Rosin Factory), mounted with neutral gum (Shanghai Aladdin Biochemical Technology Co., Ltd.), and digitally scanned using a Pannoramic 250 Flash III slide scanner (3DHistech, Budapest, Hungary).

Initially, each group had four mice for histological analysis, but final sample sizes varied by assay due to different slice quality requirements. Quantitative analyses of TH immunohistochemistry and pSer129-αSyn immunofluorescence were conducted using three mice per group. This was because one brain sample per group did not meet the quality standards for reliable analysis. In contrast, all four mice per group were included in the quantitative analysis of IBA-1 immunofluorescence. Three representative coronal sections were chosen for evaluation per animal. For TH immunohistochemical analyses, the striatal region of interest (ROI) covered the entire striatum, approximately from + 0.98 mm to + 0.14 mm relative to bregma. The SN ROI included the entire SN, spanning about − 3.00 mm to − 3.40 mm relative to bregma. In quantitative immunofluorescence analyses of pSer129-αSyn and IBA-1, the ROI was the entire ipsilateral SN. One ROI was analyzed per section for each predefined target region. Two independent investigators, blinded to group allocation, evaluated all images, and the mean of their measurements was used. Immunohistochemistry and immunofluorescence images were analyzed using ImageJ (Fiji). The average gray values of TH-positive and IBA-1-positive signals, as well as pSer129-αSyn-positive signals, were measured within the predefined ROIs.

### Transmission Electron Microscopy

For TEM, SN tissue from 2 mice per group was dissected within 1–3 min after collection to minimize mechanical disruption. Tissue blocks were immediately immersed in ice-cold primary fixative containing 2.5% glutaraldehyde and 2% paraformaldehyde in 0.1 M phosphate buffer (pH 7.4), and trimmed to approximately 1 mm³. After fixation for 4 h, samples were rinsed three times in 0.1 M phosphate buffer and post-fixed in 1% osmium tetroxide for 2 h at room temperature in the dark. Samples were then washed, dehydrated through graded ethanol followed by acetone, infiltrated with EMBed 812 resin, embedded, and polymerized at 60 °C for 48 h. Ultrathin Sects. (60–80 nm) were cut with a diamond knife on a Leica UC7 ultramicrotome, collected on formvar-coated copper grids, stained with uranyl acetate and lead citrate, and examined using a Hitachi HT7800 transmission electron microscope. Multiple fields within the SN were imaged for each animal, with particular attention to mitochondria, lysosomes, and endoplasmic reticulum (ER). For each animal, one field was acquired at a nominal magnification of 6000× from each of five ultrathin sections. TEM was performed on two mice per group, yielding five fields per mouse and ten fields per group. For semi-quantitative analysis, all acquired fields were included. Mitochondria were classified as abnormal when they showed swelling or vacuolization, disrupted or dissolved cristae, or discontinuity of the outer membrane. Lysosomal profiles were classified as abnormal when they showed marked enlargement or vacuolization, disruption of the limiting membrane, or markedly heterogeneous contents. Organelles were included only when their boundaries and internal morphology were sufficiently visible for classification. Two investigators blinded to group allocation assessed the images independently. All identifiable mitochondria and lysosomal profiles within each field were counted. Counts from the five fields were pooled within each animal, and the percentage of abnormal organelles was calculated as the total number of abnormal organelles divided by the total number of identifiable organelles of the same type. ER morphology was assessed qualitatively because ER profiles could not be counted reliably as discrete structures.

### RNA Sequencing and Bioinformatics Analysis

Total RNA was extracted from one cerebral hemisphere. RNA quality was assessed sequentially by measuring concentration and purity (A260/280) with a Nanodrop spectrophotometer, performing precise quantification using Qubit 3.0, and evaluating integrity (RIN value) on an Agilent 2100 Bioanalyzer. Samples with RIN > 7.0 were used for library construction. Poly(A)+ mRNA was enriched, fragmented, and reverse transcribed into cDNA using random hexamers. After second-strand synthesis, purification, end repair, A-tailing, adapter ligation, and size selection, the libraries were amplified by PCR. Final library quality was verified by Qubit quantification, fragment analysis, and accurate quantification via qPCR. Pooled libraries were sequenced on an Illumina platform (150 bp paired-end).

Raw reads were processed to obtain clean data, which were then aligned to the mouse reference genome (GRCm39) using HISAT2. Gene expression quantification and differential expression analysis were performed using featureCounts and DESeq2, respectively. For the targeted analysis, DESeq2-normalized counts for *Prkn*, *Gba1*, and *Lamp2* were extracted from the RNA-seq dataset for visualization and comparison among the Control, A53T, and A53T + ATO groups. Because LAMP2A is a specific isoform of LAMP2, the gene-level RNA-seq result was reported as *Lamp2* expression and was not interpreted as LAMP2A-specific transcript expression. Genes with an adjusted *p* < 0.05 and |log_2_(fold change)| > 1 were defined as differentially expressed genes (DEGs).

To evaluate coordinated changes in predefined biological pathways and processes, we performed Gene Set Enrichment Analysis (GSEA) using the GSEA software (version 4.3.3). Pre-ranked gene lists were generated based on the log_2_ fold change derived from the differential expression analysis between comparison groups. The analysis was executed against the canonical pathways and biological process gene sets from the Molecular Signatures Database (MSigDB, version 2025.1). Gene sets with a nominal *p-value* < 0.05 and a false discovery rate (FDR) < 0.25 were considered statistically significant. The normalized enrichment score (NES) was used to interpret the direction and magnitude of enrichment.

For bioinformatic analysis, we integrated multiple public databases to elucidate the molecular network. Parkinson’s disease-related genes were obtained from GeneCards (https://www.genecards.org/). Potential targets of ATO were retrieved from the Comparative Toxicogenomics Database (CTD, https://ctdbase.org/) and the SEA Search Server (https://sea.bkslab.org/). Lipid metabolism-related genes were sourced from the LIPID MAPS database (https://lipidmaps.org/). Functional enrichment analysis was conducted on the DEGs. Protein-protein interaction (PPI) networks were constructed using the STRING database and visualized with Cytoscape software (version 3.10.3). All other statistical analyses, data integration, and graphical visualizations were performed using R software (version 4.4.2).

### Molecular Docking

Molecular docking was used to compare the predicted interactions of seven commonly used statins with human αSyn. The compounds included atorvastatin, fluvastatin, lovastatin, pitavastatin, pravastatin, rosuvastatin, and simvastatin. Their three-dimensional structures were retrieved from PubChem (http://pubchem.ncbi.nlm.nih.gov), prepared in ChemBio3D Ultra 14.0 (http://www.pcsoft.com.cn/soft/195072.html), and energy-minimized with the MM2 force field to obtain the lowest-energy conformer for each ligand. The three-dimensional structure of αSyn was obtained from UniProt (https://www.uniprot.org/uniprotkb) and the Protein Data Bank (http://www.rcsb.org/). Before docking, protein structures were inspected in PyMOL 2.2.0 (http://pymol.org/) and prepared for subsequent analysis. Polar hydrogens were added using AutoDock Tools 1.5.6 (http://autodocksuite.scripps.edu/adt/), while ligand structures were hydrogenated and optimized for conformational flexibility. Docking grids were then defined in AutoDock Tools, and semiflexible docking was carried out with AutoDock using a local search algorithm. Binding free energy values were extracted from the docking output, and root mean square deviation (RMSD) values of the docked poses were calculated and visualized in PyMOL 2.2.0.

### Statistical Analysis

Unless otherwise stated, data are presented as mean ± standard deviation. Statistical analyses were performed using GraphPad Prism 9.3 software. For comparisons among multiple groups, one-way analysis of variance (ANOVA) was used, followed by Tukey’s post hoc test if homogeneity of variance was confirmed. TEM semi-quantitative data were summarized descriptively at the animal level. No inferential statistical test was performed for TEM because only two mice per group were available. *P* values less than 0.05 were considered statistically significant. Behavioral and molecular analyses were carried out blinded.

## Results

### ATO Does not Improve Motor Impairment or Nigrostriatal Pathology in A53T Mice

**Fig. 1:**
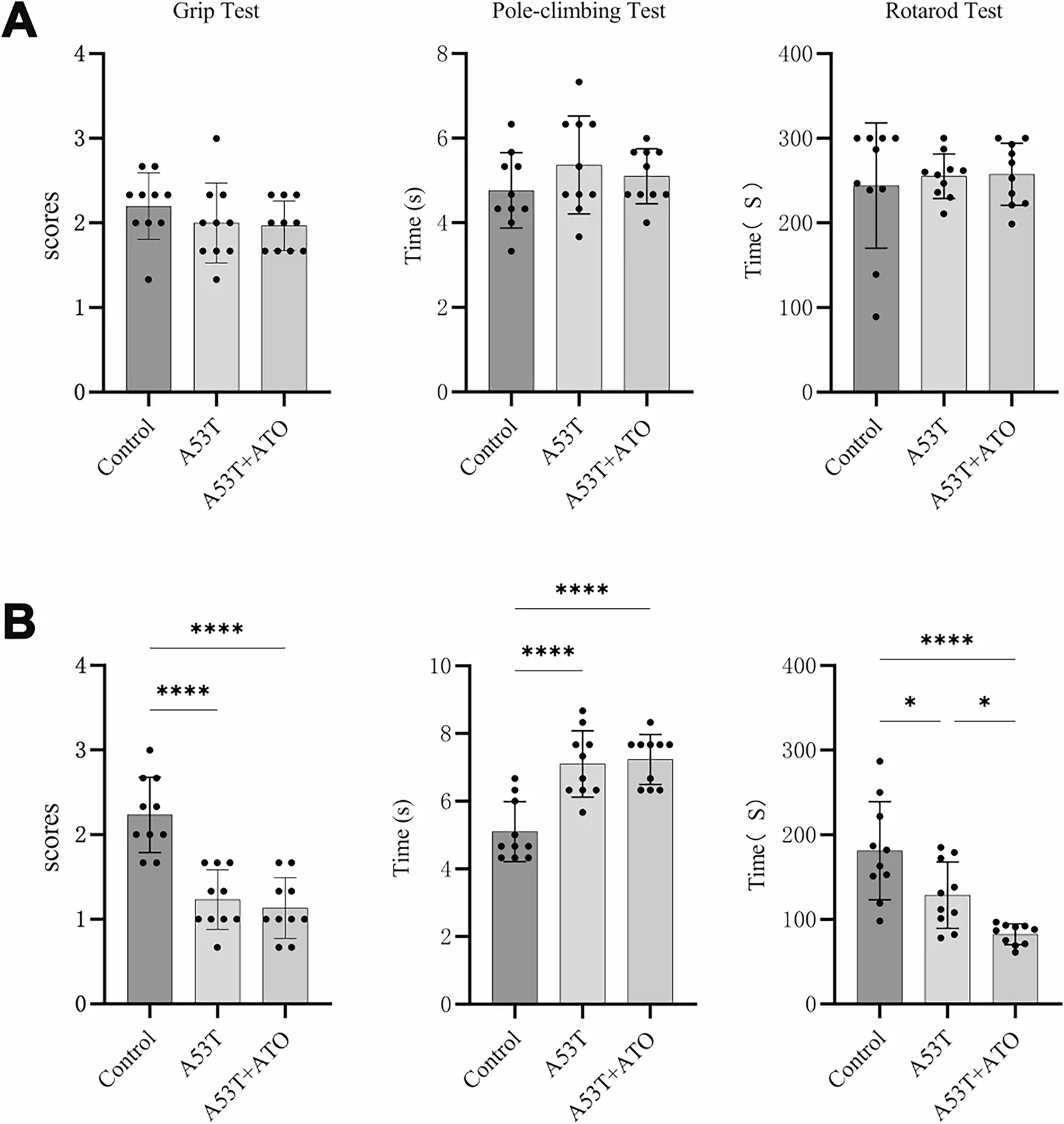
ATO treatment does not rescue motor deficits in A53T α-synuclein overexpression mice. (**A**) Baseline motor performance before treatment initiation, assessed by grip strength, pole-climbing, and rotarod test, showing no significant differences among groups. (**B**) Post-treatment motor performance. Grip strength and pole-climbing time are significantly impaired in both A53T and A53T + ATO groups compared to the control group. On the rotarod test, both A53T and A53T + ATO groups show decreased latency to fall compared with controls, with the A53T + ATO group performing significantly worse than the A53T group. Data are mean ± SD (*n* = 10 mice per group, *P < 0.05, **P < 0.01, ***P < 0.001, ****P < 0.0001 by one-way ANOVA with Tukey’s post hoc test)

To evaluate the therapeutic potential of ATO in a genetic model of PD, we used a well-established murine model in which rAAV9 encoding human A53T-mutant αSyn was bilaterally injected into the SN of C57BL/6 mice recapitulates key features of PD. Mice were divided into three groups: Control (empty rAAV9 vector-injected), A53T (rAAV9-A53T-injected), and A53T + ATO (rAAV9-A53T-injected and ATO-treated). Motor functions were assessed before and after treatment. Baseline grip strength, pole-climbing, and rotarod test performance did not differ among groups (Fig. 1A), confirming a consistent baseline. After treatment, motor deficits appeared in the A53T group. Grip strength and pole-climbing performance were significantly impaired in both A53T and A53T + ATO groups compared with controls, with no significant difference between the two disease groups (Fig. 1B). The rotarod test showed a graded deficit: both A53T and A53T + ATO groups showed lower latency to fall than controls, and the A53T + ATO group performed significantly worse than the A53T group (Fig. 1B).

**Fig. 2:**
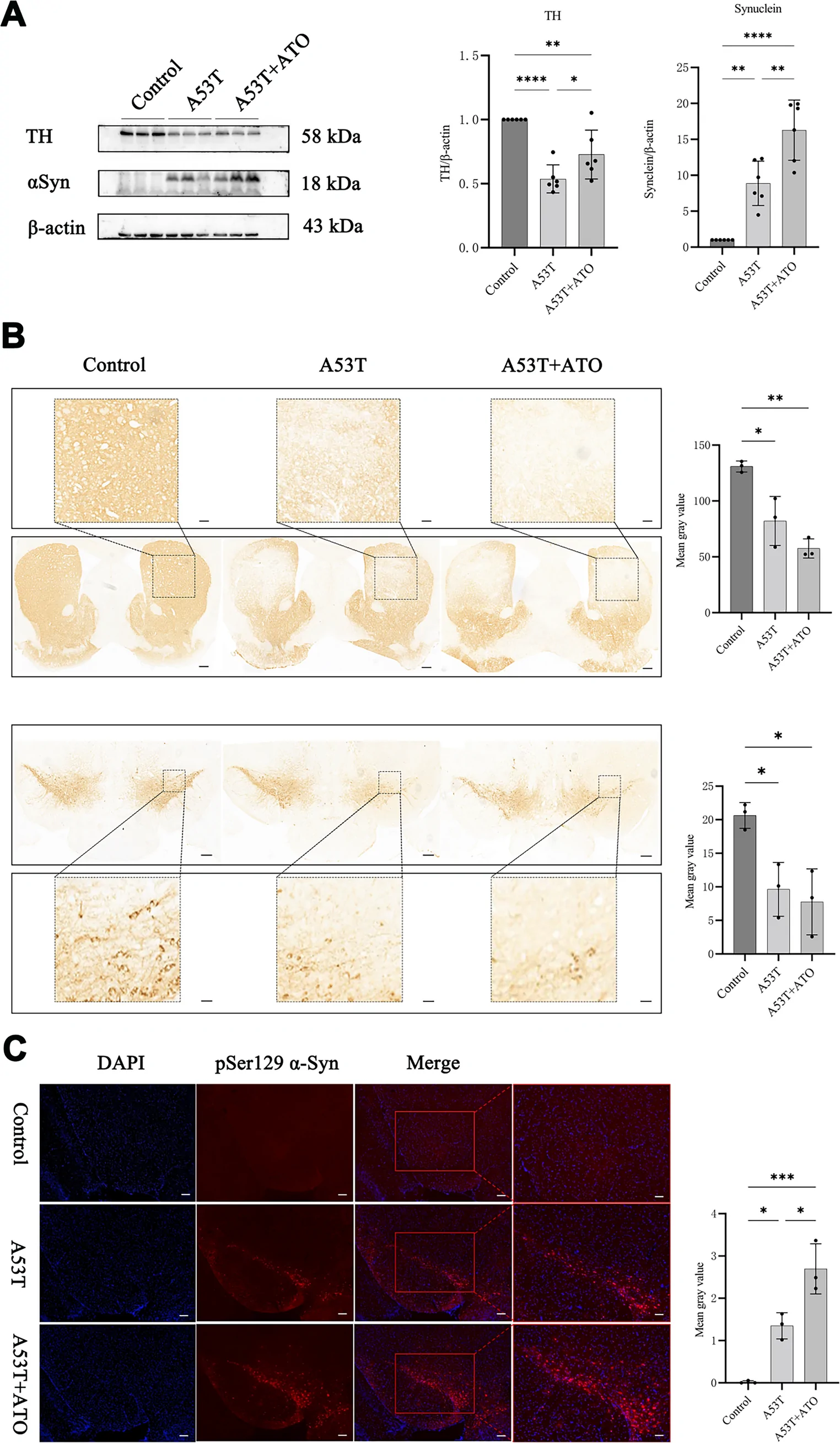
ATO does not restore TH-related measures or reduce αSyn-related signals in A53T mice. (**A**) Western blotting of TH and αSyn in SN RIPA lysates. TH expression was significantly reduced, whereas αSyn levels were increased in both the A53T and A53T + ATO groups compared with controls. TH expression tended to be lower, and the αSyn signal was numerically highest, in the A53T + ATO group (*n* = 6 mice per group). (**B**) Representative TH immunohistochemistry in the striatum (upper panels) and SN (lower panels). TH-positive signals were reduced in both the A53T and A53T + ATO groups compared with controls (*n* = 3 mice per group). Striatum, scale bars: 500 μm and 166.7 μm (3× magnified views); SN, scale bars: 200 μm and 33.3 μm (6× magnified views). (**C**) Immunofluorescence staining for pSer129-αSyn (red) in the SN. Increased pSer129-αSyn immunoreactivity was observed in A53T mice and remained elevated in the A53T + ATO group (*n* = 3 mice per group). Scale bars: 200 μm and 100 μm (2× magnified views). Data are mean ± SD (*P < 0.05, **P < 0.01, ***P < 0.001, ****P < 0.0001 by one-way ANOVA with Tukey’s post hoc test)

Consistent with the behavioral deficits, western blotting of SN RIPA lysates showed significantly reduced TH expression and increased αSyn levels in both the A53T and A53T + ATO groups compared with controls. TH reduction tended to be greater, and the αSyn signal was numerically highest, in the A53T + ATO group (Fig. 2A). TH immunohistochemistry showed reduced TH-positive signals in the striatum and SN of both disease groups (Fig. 2B). pSer129-αSyn immunofluorescence was increased in the SN of A53T mice and was significantly higher in the A53T + ATO group than in the A53T group (Fig. 2C). ATO did not improve motor deficits, restore TH-related measures, reduce αSyn levels in RIPA lysates, or decrease pSer129-αSyn immunoreactivity in the A53T αSyn overexpression model.

### ATO Alters Microglial Morphology While Ultrastructural Abnormalities Remain Evident

**Fig. 3:**
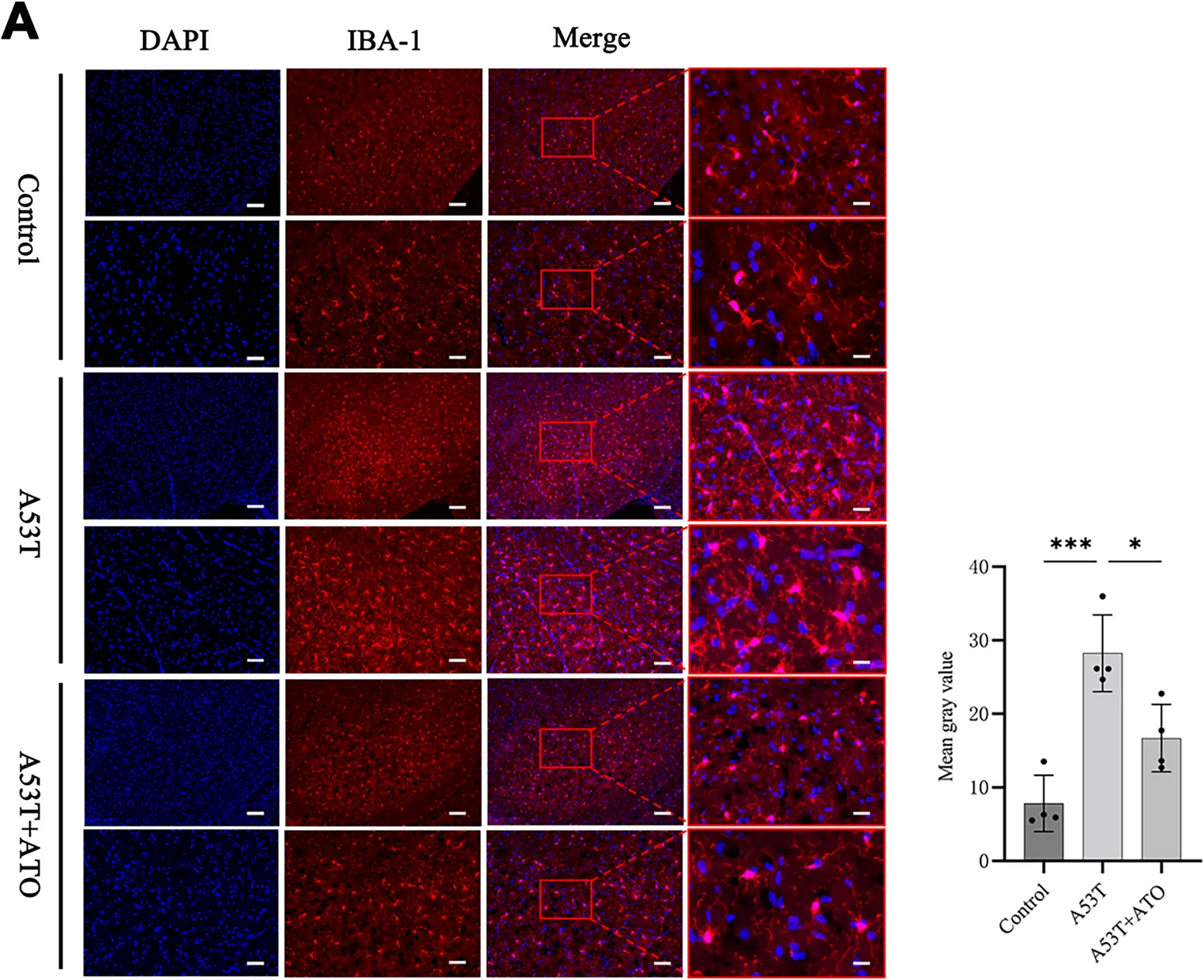

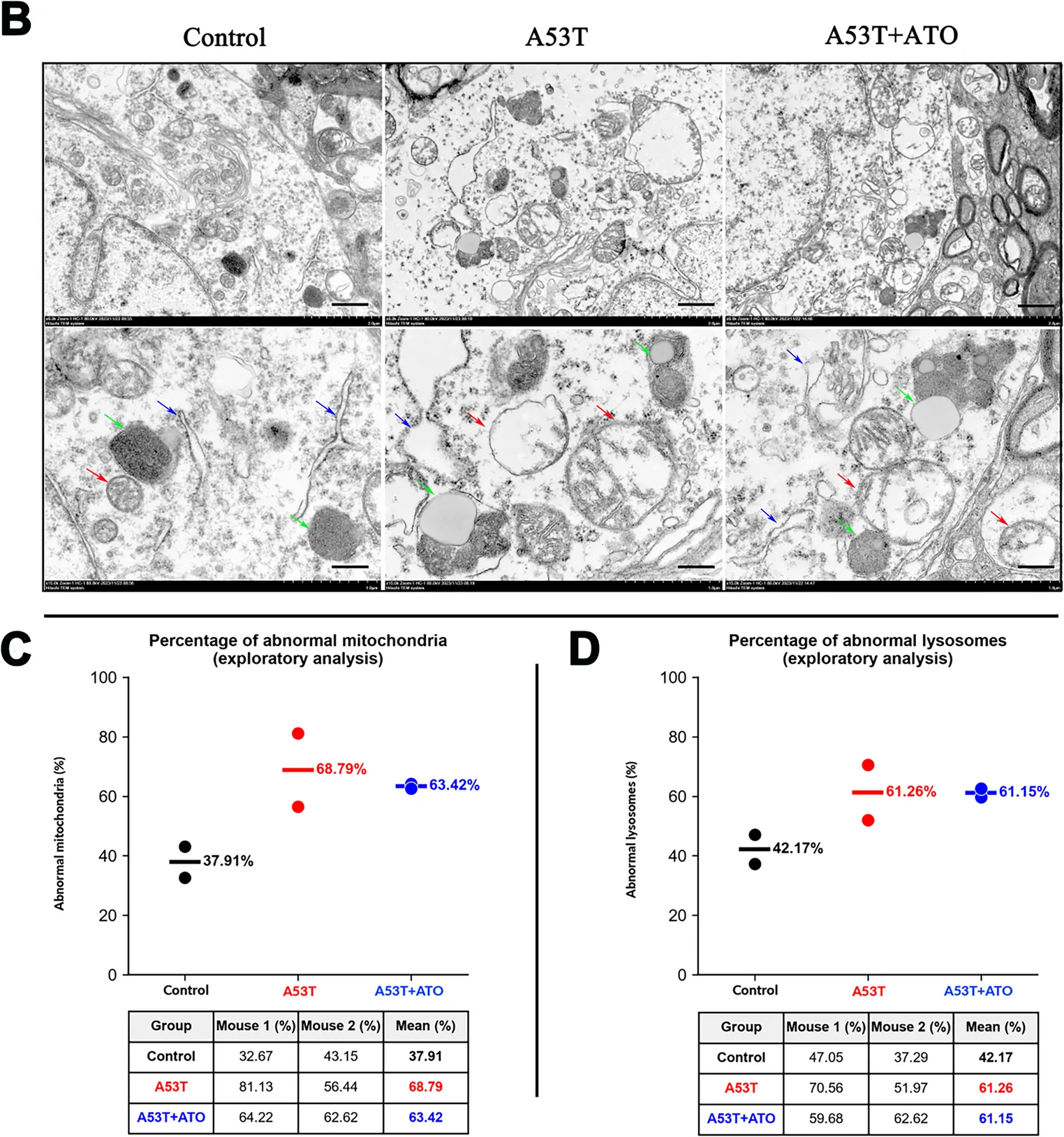
Effects of ATO on microglial morphology and nigral ultrastructure. (**A**) Representative immunofluorescence images of IBA-1⁺ microglia in the SN (left) and quantitative analysis of IBA-1 immunoreactive signal intensity (right). Microglia in control mice exhibit a highly ramified, surveillant morphology. In A53T mice, microglia show an activated phenotype with retracted, thickened processes and enlarged cell bodies, accompanied by increased IBA-1 immunoreactive signal intensity. The A53T + ATO group displays a heterogeneous microglial phenotype with intermediate morphological features and altered IBA-1 immunoreactive signal intensity compared with the A53T group (*n* = 4 mice per group). Scale bars: 200 μm and 50 μm (4× magnified views) (**B**) Representative TEM images of nigral neurons. Control neurons display normal organelles. A53T neurons exhibit severe organelle damage including vacuolated mitochondria with dissolved cristae (red arrows), abnormal lysosomes with heterogeneous content (green arrows), and dilated ER (blue arrows). These pathological features persist in the A53T + ATO group. Representative images are shown from 2 mice per group. Scale bars: 4 μm (**C**,** D**) Exploratory semi-quantitative analysis of abnormal mitochondria and lysosomes in the available TEM images. Counts from the five fields were pooled within each animal, and animal-level percentages are shown. Each point represents one mouse. TEM was performed on two mice per group, and no inferential statistical testing was conducted

We next investigated the effect of ATO on microglial morphology and subcellular organelle integrity. Analysis of IBA-1 immunofluorescence in the SN revealed distinct microglial activation profiles (Fig. 3A). Control microglia exhibited a highly ramified, surveillant morphology with fine, dense processes. In contrast, A53T mice displayed a highly activated phenotype, characterized by retracted, thickened processes and enlarged amoeboid cell bodies. The A53T + ATO group presented a heterogeneous state, featuring a mixture of cells with intermediate activation (some ramified, others with thickened processes) and reduced IBA-1 immunoreactive signal intensity.

We then performed TEM to assess ultrastructural morphology in neuronal profiles within the SN (Fig. 3B). Control samples showed comparatively preserved mitochondrial membranes and cristae, more uniform lysosomal contents, and better-organized ER cisternae. A53T samples showed mitochondrial swelling or vacuolization, disrupted cristae, heterogeneous or vacuolated lysosomal profiles, and dilated or disorganized ER cisternae. Similar abnormalities remained visible in the A53T + ATO group.

Semi-quantitative analysis included five fields from five ultrathin sections per mouse. Counts from the five fields were pooled within each animal. The percentages of abnormal mitochondria were 32.67% and 43.15% in the two Control mice, 81.13% and 56.44% in the two A53T mice, and 64.22% and 62.62% in the two A53T + ATO mice, with group means of 37.91%, 68.79%, and 63.42%, respectively. The corresponding percentages of abnormal lysosomes were 47.05% and 37.29% in the Control group, 70.56% and 51.97% in the A53T group, and 59.68% and 62.62% in the A53T + ATO group, with group means of 42.17%, 61.26%, and 61.15%, respectively. Individual animal values are shown in Fig. 3C and D.

Mitochondrial and lysosomal abnormalities were more frequent in A53T samples than in Control samples and remained evident after ATO treatment. The A53T + ATO group did not show a clear reduction relative to the A53T group. Because TEM was performed on two mice per group, these findings were interpreted descriptively without inferential statistical testing.

### ATO Modulates Cholesterol-Related Transcriptional Programs but Fails to Reverse the Disease Core Signature

**Fig. 4:**
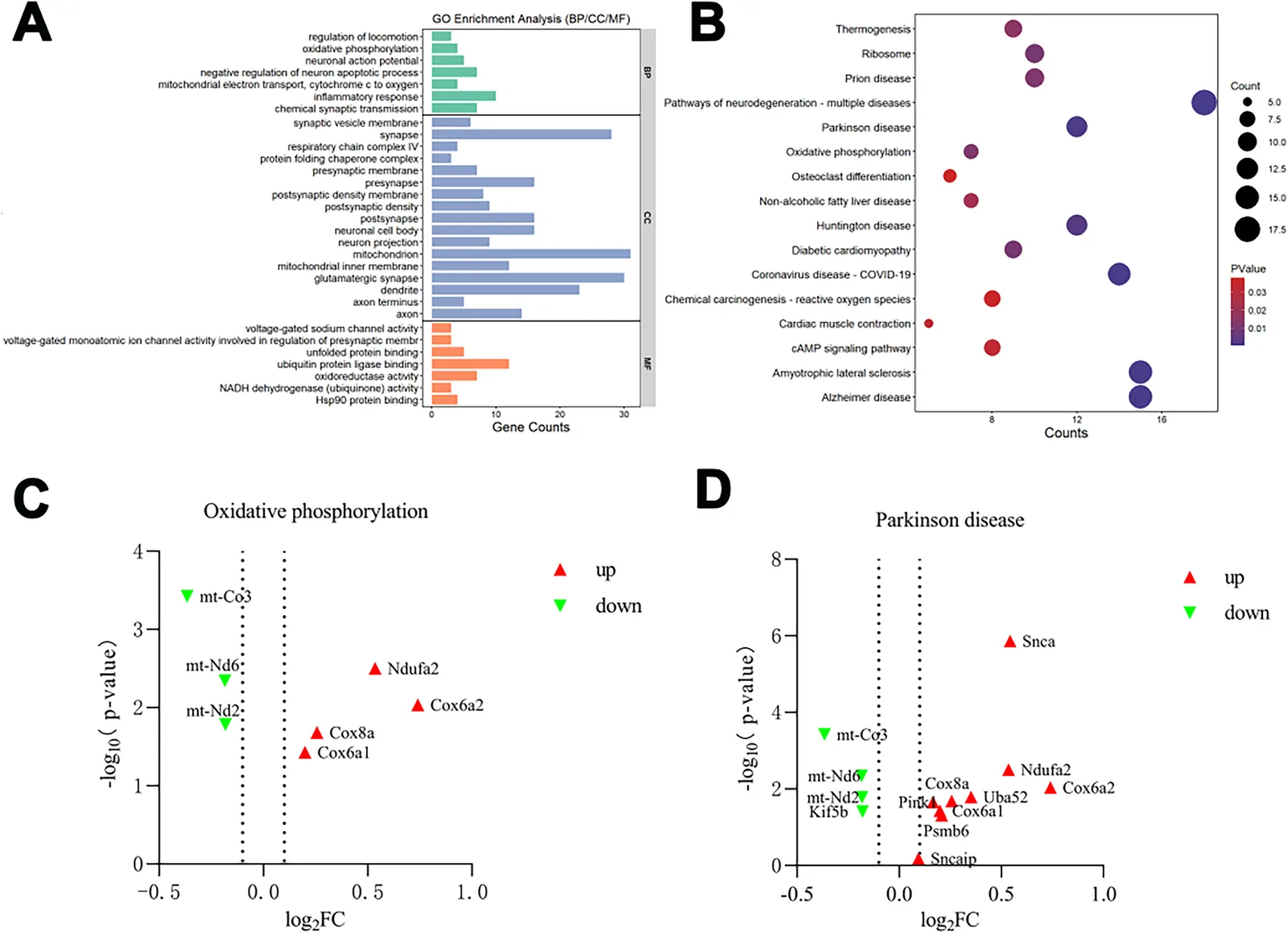
Transcriptomic profiling confirms PD-like pathophysiology in A53T α-synuclein overexpression mice. (**A**) GO enrichment analysis of DEGs between A53T and control groups, categorized by Biological Process, Cellular Component, and Molecular Function. Key terms related to mitochondrial function, synaptic transmission, locomotion, protein folding, inflammation, and chaperone activity are highlighted. (**B**) KEGG pathway enrichment analysis for the A53T vs. control comparison, showing significant enrichment for pathways involved in neurodegeneration (including Parkinson’s disease and Alzheimer’s disease), amyotrophic lateral sclerosis, and oxidative phosphorylation. (**C**,** D**) Volcano plots depicting DEGs within the (C) oxidative phosphorylation and (D) Parkinson’s disease KEGG pathways in A53T mice compared with controls. Significantly upregulated and downregulated genes are shown in red and blue, respectively (|log₂ (fold change)| > 1, adjusted *P* < 0.05)

To gain a systems-level understanding, we performed RNA sequencing on the brain tissue. Comparison of Control and A53T groups supported the presence of PD-related molecular perturbations in the model. Gene Ontology (GO) enrichment showed significant perturbations in biological processes important for PD such as oxidative phosphorylation, mitochondrial electron transport, chemical synaptic transmission, locomotion regulation, protein folding and inflammatory response (Fig. 4A). Cellular components showed significant alterations in mitochondrial, synaptic (presynaptic, postsynaptic, vesicle), neuronal structure (axon, dendrite, cell body) compartments, protein folding chaperone complex (Fig. 4A). Molecular function terms were rich in NADH dehydrogenase activity, oxidoreductase activity, voltage-gated ion channel activity, unfolded protein binding, Hsp90 binding and ubiquitin ligase binding (Fig. 4A). KEGG pathway analysis confirmed the validity of the model. The model showed significant enrichment of neurodegeneration-related pathways, Parkinson’s disease, Alzheimer’s disease, amyotrophic lateral sclerosis and oxidative phosphorylation pathways (Fig. 4B). Volcano plots for genes in oxidative phosphorylation and Parkinson’s disease pathways showed widespread dysregulation in the A53T model (Figs. 4C, D).

**Fig. 5:**
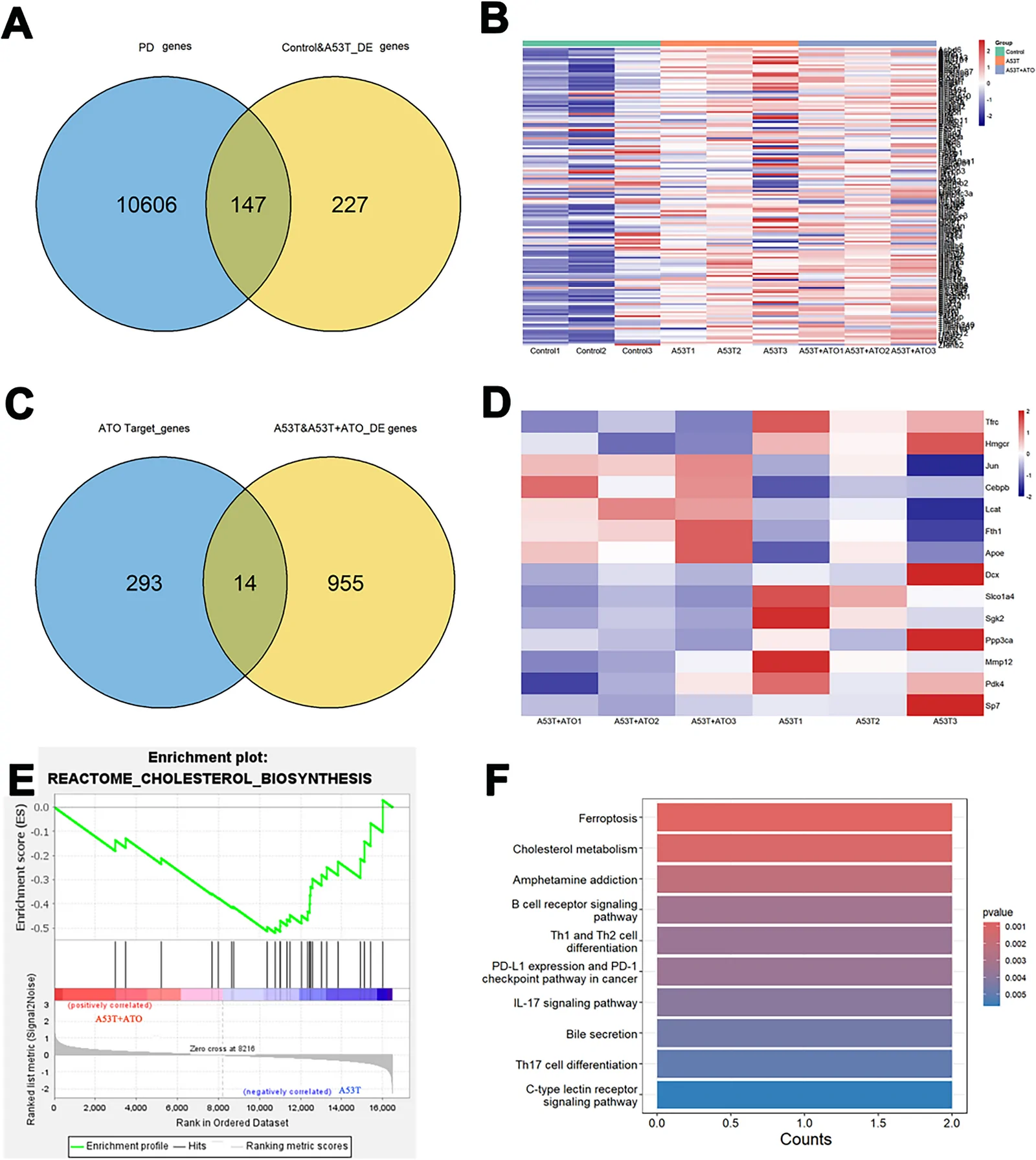
ATO modulates statin-associated transcriptional programs but fails to reverse the core PD molecular signature. (**A**) Venn diagram illustrating the identification of a DCS. The intersection of DEGs between A53T and control groups with a curated list of human Parkinson’s disease-associated genes from GeneCards yielded 147 DCS genes. (**B**) Heatmap of the 147 DCS genes across the three experimental groups. The signature is robustly activated in the A53T group and remains elevated in the A53T + ATO group. (**C**) Venn diagram identifying 14 putative ATO-target genes via the intersection of DEGs between A53T and A53T + ATO groups with known statin-target gene sets. (**D**) Heatmap confirming the expression modulation of the 14 putative ATO-target genes in response to treatment. (**E**) GSEA plot showing significant downregulation of the “REACTOME_CHOLESTEROL_BIOSYNTHESIS” pathway in the A53T + ATO group compared to the A53T group, consistent with a canonical statin-responsive transcriptional effect (NES: −1.92; FDR: 0.0036; *P* < 0.05). (**F**) KEGG pathway analysis of the 14 ATO-target genes, indicating their involvement in cholesterol metabolism, ferroptosis, and other pathways

We next defined a disease core signature (DCS) by intersecting the DEGs from the A53T versus control comparison with a curated list of human PD-associated genes from GeneCards, which yielded 147 genes (Fig. 5A). Heatmap showed that the DCS was activated robustly in A53T group and was persistently high in A53T + ATO group (Fig. 5B), suggesting that ATO failed to reverse the core disease signature.

To characterize transcriptional changes associated with statin-responsive pathways, we identified 14 putative ATO-associated genes by intersecting DEGs between the A53T and A53T + ATO groups with known statin-associated gene sets (Fig. 5C). Heatmap profiling of these targets indicated possible activation of these genes in response to ATO intervention (Fig. 5D). GSEA of all DEGs showed significant downregulation of the REACTOME_CHOLESTEROL_BIOSYNTHESIS pathway in ATO-treated mice (Fig. 5E), consistent with previously described downstream transcriptional effects associated with statin treatment. KEGG analysis of 14 ATO-target genes showed that they play key roles in cholesterol metabolism and ferroptosis (Fig. 5F).

### ATO Induces Lipid-Centered Reprogramming Accompanied by Increased SNCA Expression

**Fig. 6:**
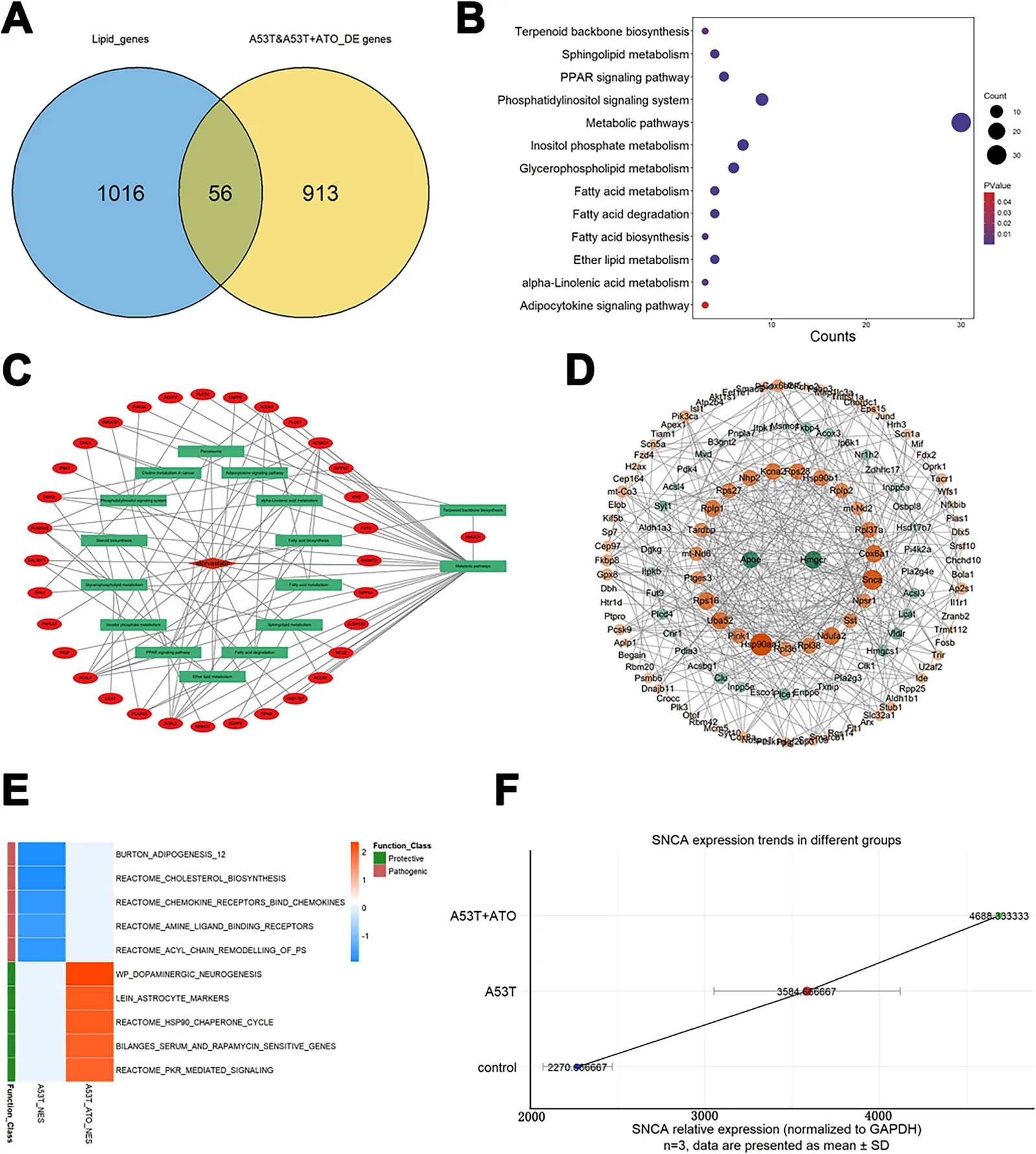
ATO induces lipid-related transcriptional changes that are accompanied by persistent SNCA upregulation Venn diagram identifying 56 “ATO-responsive lipid genes” from the intersection of DEGs between the A53T and A53T + ATO groups and a lipid gene database. (**B**) KEGG pathway enrichment analysis of the 56 ATO-responsive lipid genes shows concerted enrichment in diverse lipid metabolic pathways. (**C**) Protein-protein interaction network (based on STRING database) illustrating connectivity among the ATO-responsive lipid genes, ATO, and the enriched KEGG pathways. (**D**) A separate interaction network demonstrates significant physical interactions between the ATO-perturbed lipid module and proteins within the Disease Core Signature. (**E**) GSEA reveals bidirectional functional remodeling induced by ATO, downregulating several pathogenic pathways while concurrently upregulating putative protective pathways (|NES| > 1, FDR < 0.25, *P* < 0.05). (**F**) Quantitative analysis of *SNCA* mRNA expression. Levels are significantly higher in the A53T + ATO group compared to the A53T group, concomitant with the observed increase in αSyn protein (see Fig. 2A)

We next examined whether ATO induced a lipid-centered molecular response distinct from the core disease program. We identified 56 “ATO-responsive” lipid genes linking the DEGs between the A53T and the A53T + ATO groups with a database of lipid genes (Fig. 6A). KEGG analysis showed that these lipid-related genes were enriched in multiple pathways (Fig. 6B). Protein-protein interaction network analyses showed that the ATO-responsive lipid genes were connected to ATO-associated pathways and to components of the disease core signature (Fig. 6C, D). GSEA further indicated a bidirectional pattern, with downregulation of several pathways associated with injury and upregulation of others with potentially protective implications (Fig. 6E). Despite these changes, *SNCA* mRNA expression was significantly higher in the A53T + ATO group than in the A53T group (Fig. 6F), consistent with the increase in αSyn protein observed by western blotting (Fig. 2A).

### ATO-Associated SNCA Upregulation is not Accompanied by Increased Expression of αSyn Clearance-Related Markers

**Fig. 7:**
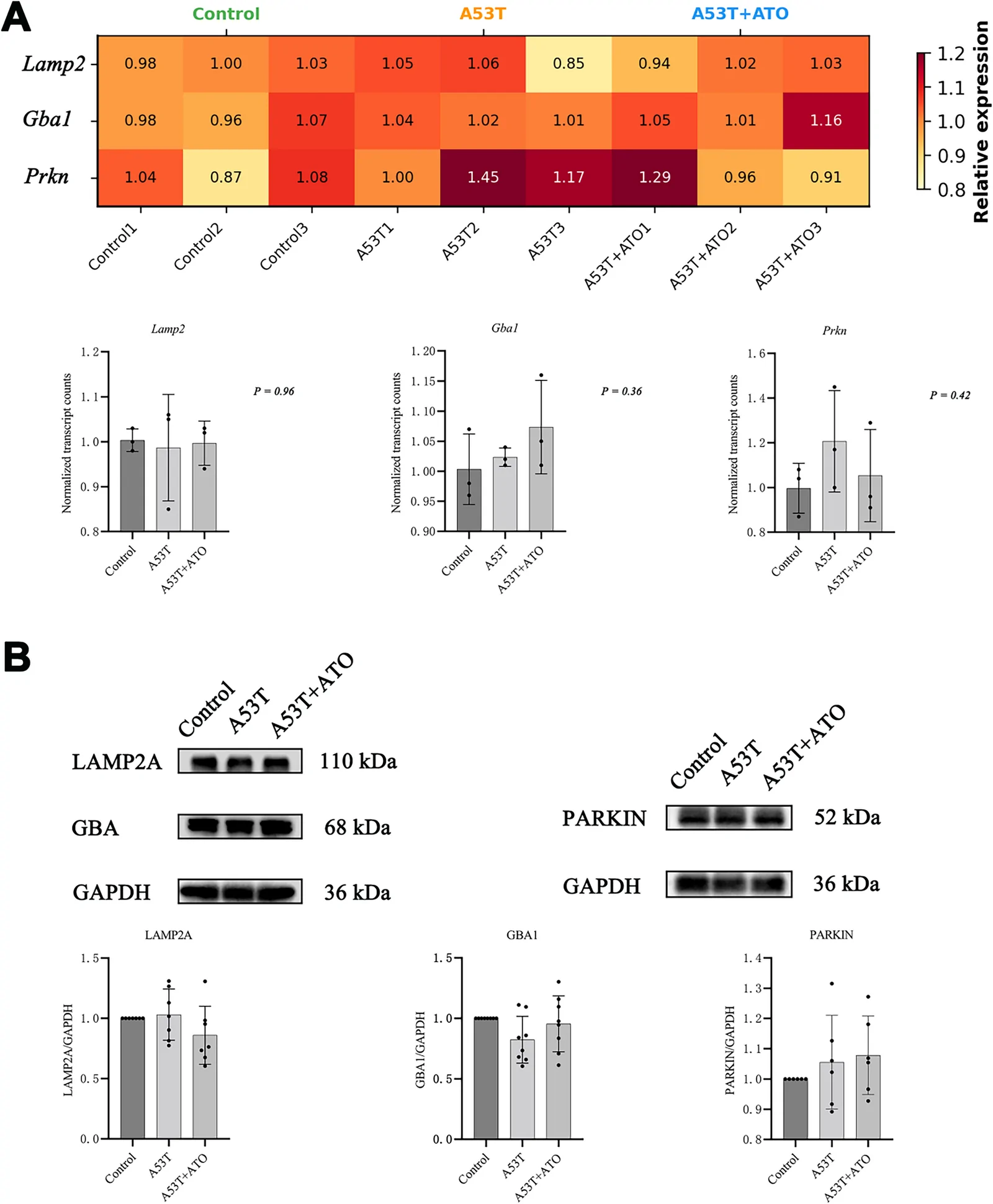
αSyn clearance-related markers remain unchanged after ATO treatment Heatmap and quantification of *Prkn*, *Gba1*, and *Lamp2* transcript levels in individual RNA-seq samples from the Control, A53T, and A53T + ATO groups. The three groups showed no significant differences (*n* = 3 mice per group) Representative western blots and densitometric quantification of PARKIN, GBA1, and LAMP2A protein expression. Protein levels were normalized to GAPDH. No significant differences were detected among the three groups (*n* = 6 mice per group). Data are presented as mean ± SD. Group comparisons were performed using one-way ANOVA followed by Tukey’s post hoc test

Because *SNCA* mRNA increased after ATO treatment while αSyn protein burden remained elevated, we examined *Prkn*, *Gba1*, and *Lamp2* transcript abundance and PARKIN, GBA1, and LAMP2A protein expression. Targeted RNA-seq analysis showed no significant differences in *Prkn*, *Gba1*, or *Lamp2* transcript abundance among the Control, A53T, and A53T + ATO groups (Fig. 7A). Because gene-level RNA-seq does not distinguish the LAMP2A isoform, *Lamp2* was reported at the gene level. Western blot analysis also showed no significant differences in PARKIN, GBA1, or LAMP2A protein expression among the three groups (Fig. 7B). None of these proteins increased significantly in the A53T + ATO group relative to the A53T group. The ATO-associated increase in *SNCA* mRNA and persistent αSyn protein burden were therefore not accompanied by higher expression of the examined clearance-related markers.

### Statins Show Differential Predicted Binding Profiles with αSyn

**Fig. 8:**
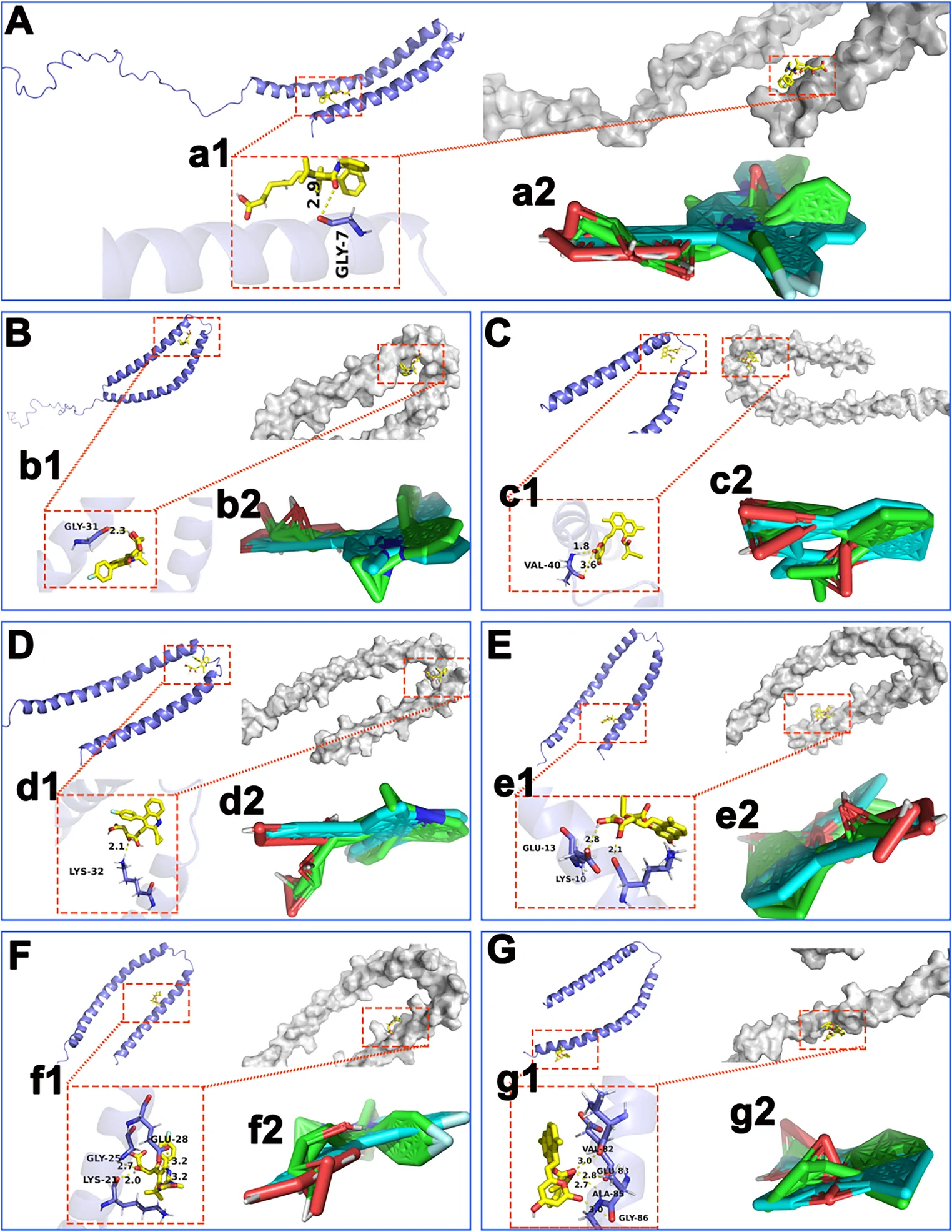
Exploratory molecular docking comparison of seven statins with human αSyn.**(A) **Atorvastatin. (**B) **Fluvastatin. (**C) **Lovastatin. (**D) **Pitavastatin. (**E) **Pravastatin. (**F) **Rosuvastatin. (**G) **Simvastatin (a1–g1) Molecular docking results are presented in two-dimensional and three-dimensional formats, sequentially showing the docking profiles of αSyn with each of the seven statins (a2-g2) 3D structural representations depicting the differences in molecular conformations between the docked and reference conformations of αSyn with each of the seven statins

To complement the pathway-level analyses, we performed molecular docking as an exploratory structural comparison of seven commonly used statins with human αSyn (Fig. 8; Table 1). The analysis included predicted binding free energy (ΔG), RMSD, inhibitory constant (Ki), and hydrogen-bonding patterns. Across the compounds tested, the docking results showed clear differences in predicted interaction profiles. ATO showed a predicted binding free energy of − 0.79 kcal/mol, an RMSD of 1.051 Å, a Ki of 265.73 mM, and one hydrogen bond involving GLY-7. Lovastatin, simvastatin, and pitavastatin showed lower predicted binding energies, at − 2.40, − 2.59, and − 2.74 kcal/mol, respectively, together with lower Ki values than ATO. Simvastatin formed four hydrogen bonds, whereas pitavastatin formed one hydrogen bond with LYS-32. Rosuvastatin showed an intermediate predicted binding energy (− 1.17 kcal/mol) with a relatively higher RMSD (1.253 Å), while pravastatin showed minimal predicted binding in this analysis (0.14 kcal/mol). Overall, the docking analysis indicated non-uniform predicted statin-αSyn interaction patterns across compounds.

**Table 1:** Molecular docking results of seven statins against human αSyn

Statin	Binding Energy (kcal/mol)	RMSD (Å)	Inhibitory Constant (kI)	Hydrogen Bonds (Number & Key Residues)
Atorvastatin	−0.79	1.051	265.73 mM	1 (GLY-7)
Fluvastatin	−1.96	0.763	36.42 mM	1 (GLY-31)
Lovastatin	−2.40	0.560	17.29 mM	2 (VAL-40)
Pitavastatin	−2.74	0.824	9.77 mM	1 (LYS-32)
Pravastatin	0.14	0.674	unavailable	2 (GLU-13, LYS-10)
Rosuvastatin	−1.17	1.253	139.88 mM	4 (GLY-25, LYS-21, GLU-28)
Simvastatin	−2.59	0.695	12.65 mM	4 (VAL-82, GLU-83, ALA-85, GLY-86)

## Discussion

In this study, ATO altered lipid-related transcriptional programs in the A53T αSyn overexpression mouse model, but these changes did not translate into clear functional or neuropathological benefit. Treatment did not improve grip strength or pole-climbing performance. It also did not restore TH expression, lower αSyn levels in RIPA lysates, or reduce pSer129-αSyn immunoreactivity. Rotarod performance was worse in the A53T + ATO group than in untreated A53T mice. These findings indicate limited benefit of ATO in this αSyn-driven setting.

The lack of a beneficial effect of ATO should be interpreted in the context of the underlying pathological phenotype. If the baseline pathological alterations are mild or inconsistent, it is difficult to determine whether the drug truly failed to rescue the phenotype. In our study, A53T mice developed motor abnormalities, reduced TH-related measures, increased αSyn-related signals, organelle abnormalities, and broad transcriptional changes in processes relevant to PD. These endpoints provided a measurable range in which a treatment effect could have been detected. The model nevertheless has a restricted scope. AAV-mediated A53T αSyn overexpression produces a localized and accelerated insult that does not reproduce the slow progression or anatomical spread of human PD. The assays used in this study did not establish mature Lewy body-like pathology. The present findings apply to this αSyn-driven experimental setting and should not be generalized to PD as a whole. Future studies using complementary PD models, including αSyn seeding-based paradigms and chronic progressive models, will be important to determine whether the response to ATO depends on disease stage, αSyn propagation, or the underlying pathogenic context.

This pattern does not fully match reports from toxin-based PD models, in which some statins have shown protective effects (Kumar et al. 2012; Marques et al. 2018; Yan et al. 2020a). The difference is likely related to the type of injury driving each model. Toxin models such as MPTP mainly reflect acute oxidative and inflammatory stress (Huang et al. 2023; Zhang et al. 2022). Anti-inflammatory and potentially mitochondrial-protective effects of statins are therefore more likely to be beneficial in such models (Arendt et al. 2025; Morel et al. 2017). ATO has been shown to improve behavioral deficits in MPTP-treated mice by modulating NADPH oxidase 2 (NOX2)-mediated oxidative stress and autophagy pathways (Yan et al. 2020a). Other studies have shown that the drug can reduce early oxidative events and reconstruct the network of inflammatory mediators in the striatum after MPTP exposure (Marques et al. 2018). However, our model is driven by sustained A53T αSyn expression in the SN, which places continuous stress on protein homeostasis, synaptic function, and neuronal survival (Karikari et al. 2022; Kirik et al. 2002; Theodore et al. 2008; Volpicelli-Daley et al. 2011). This context may be closer to PD subtypes linked to *SNCA* mutation or overexpression. In such a setting, changing part of the downstream response may still fail to alter the main disease course. Meanwhile, the glial and neuronal findings should also be considered together. After ATO treatment, microglia transitioned from a uniformly activated, amoeboid form toward an intermediate phenotype, indicating that ATO modulates local inflammation-related responses. Neuronal ultrastructural damage, however, did not improve in parallel: mitochondria remained swollen and damaged, lysosomal abnormalities persisted, and the ER remained disorganized. These observations imply that partial modulation of microglial state alone is insufficient to mitigate ongoing αSyn-related cellular stress. These results align with clinical studies reporting mixed associations between statin use and PD prevention or disease modification, and they do not provide definitive evidence of neuroprotection (Mady et al. 2024).

Transcriptomic analyses provide a plausible alternative explanation for ATO’s lack of efficacy. After ATO treatment, transcriptional programs related to cholesterol biosynthesis and lipid metabolism changed in brain tissue. This pattern indicates a treatment-associated molecular response but does not establish atorvastatin concentration or therapeutically sufficient CNS exposure. Although prior studies show that statins can reshape broad transcriptional networks, including sterol-regulatory pathways (Shi and Han 2025), it remains uncertain whether they directly regulate *SNCA* transcription in the setting of αSyn pathology. In *vitro* experiments have suggested that statins reduce αSyn aggregation (Bar-On et al. 2008), while recent human brain tissue models indicate that isolated disruptions of cholesterol metabolism may promote αSyn pathology (Mesentier-Louro et al. 2025). Given these mixed findings, it is perhaps unsurprising that interventions targeting lipid metabolism yield inconsistent outcomes across models. In the present A53T model, however, lipid-focused interventions failed to meaningfully reverse the disease-associated core transcriptional signatures. In parallel, *SNCA* mRNA did not decline; it increased, consistent with the observed rise in αSyn protein levels in treated animals. These results do not demonstrate that ATO directly upregulates *SNCA* transcription, and causality should not be inferred. Rather, the collective data suggest that the molecular changes in lipid metabolism induced by ATO did not redirect the αSyn-centered disease program toward a less pathogenic state. Put differently, ATO appears to have provoked a molecular response that accompanied disease progression instead of counteracting its central pathological features.

αSyn homeostasis depends on the balance between its production and clearance. (Ramos Dos Santos et al. 2026) reported that PRKN links the regulation of *SNCA* expression to GBA1- and LAMP2A-associated chaperone-mediated autophagy. This coordination was impaired in cellular and mouse models of Parkinson’s disease and in affected human brain tissue. LAMP2A serves as the lysosomal receptor for chaperone-mediated autophagy, while GBA1 supports lysosomal function and αSyn turnover (Cuervo et al. 2004; Mazzulli et al. 2011). We therefore examined whether the increase in *SNCA* expression was accompanied by changes in PRKN, GBA1, and LAMP2A. However, transcript levels of *Prkn*,* Gba1*, and *Lamp2* showed no increase. PARKIN, GBA1, and LAMP2A protein expression also remained unchanged. The absence of a parallel response in these clearance-related markers may partly explain the persistent αSyn protein burden after ATO treatment. Notably, the expression data showed no detectable compensatory upregulation, although αSyn clearance itself was not directly measured.

The mismatch between drug-induced molecular changes and observable phenotypic benefit persists across pathway, network, and molecular-structure scales. GSEA did not show a single-direction deleterious or protective signature. Instead, some injury-associated pathways were downregulated while certain potentially protective pathways were upregulated. Although such bidirectional modulation can be beneficial in other settings, the dominant αSyn-driven pathology in our model remained unchanged, making it unlikely that these opposing shifts would produce a meaningful net benefit. Protein–protein interaction network analysis reinforces this conclusion. The ATO-responsive lipid module was linked to the disease core network, indicating that the drug-induced alterations occurred within molecular networks relevant to the central pathological process rather than in an unrelated peripheral background. Nevertheless, these network-level transcriptional changes were not accompanied by improvement of the core behavioral or neuropathological phenotypes. To explore possible molecular explanations, we used molecular docking to evaluate whether predicted differences in statin–αSyn interactions could account for the disconnect between molecular changes and phenotype. These docking results are presented strictly as supplementary structural clues rather than standalone proof and should therefore be interpreted with caution. Our docking analysis predicts only weak binding between ATO and αSyn (ΔG = − 0.79 kcal/mol), whereas lovastatin, simvastatin, and pitavastatin show substantially stronger predicted affinities. This pattern aligns with earlier reports of lovastatin’s beneficial effects in α-synucleinopathy models (Koob et al. 2010), but docking alone neither demonstrates in vivo target engagement nor establishes that direct statin–αSyn binding is required for neuroprotection. Taken together, the molecular docking data offer a plausible structural explanation for the differing efficacy of statins in α-synucleinopathy models. When integrated with the transcriptomic results, they support a consistent conclusion: under the conditions of the present study, ATO is biologically active, yet its activity does not meaningfully modify the principal pathogenic process in this model.

Several features limit the scope of this study. The AAV-A53T model produces an accelerated and anatomically restricted αSyn-driven pathology that does not fully capture the progressive nature of human Parkinson’s disease, and the assays used here did not distinguish detergent-soluble from insoluble αSyn species or establish mature Lewy body-like pathology. ATO was tested at one dose beginning one day after vector delivery, overlapping with disease development rather than established pathology, and its concentrations in plasma and brain tissue were not measured. The increase in *SNCA* expression was evaluated together with clearance-related markers, but these measurements do not directly assess αSyn turnover or lysosomal function. The relatively limited cohorts used for histological, ultrastructural, and transcriptomic analyses may have reduced sensitivity for detecting subtle biological changes, particularly in region-specific molecular alterations. In addition, only male mice were examined, and potential sex-dependent responses remain to be determined. These constraints define the context in which the findings should be interpreted: ATO altered lipid-related transcriptional programs but did not produce measurable behavioral or neuropathological improvement under the conditions tested.

## Conclusion

At the tested dose and treatment schedule, ATO was biologically active in the AAV-A53T αSyn mouse model, but did not lead to functional or histopathological benefit. Transcriptomic profiling showed substantial modulation of cholesterol- and lipid-related programs, whereas the main αSyn-driven disease signature changed little. Molecular docking suggested a relatively weak predicted interaction between ATO and αSyn. This provides possible context, but not proof, for why the detectable brain-tissue pharmacodynamic response failed to translate into measurable neuroprotection in this model. These findings indicate that ATO has limited disease-modifying effects in this αSyn-driven model and should be further evaluated in complementary PD models representing different stages of disease progression.

## Supplementary Information

Below is the link to the electronic supplementary material.

Supplementary Material 1 (DOCX 7.01 MB)

Supplementary Material 2 (ZIP 1.89 MB)

## Data Availability

All data collections employed and analyzed for the purposes of this research are available from the corresponding author on request from qualified investigators.

## References

[CR1] Arendt N, Kopsida M, Lennernäs H, Sjöblom M, Heindryckx F (2025) Statin-mediated protection in chemotherapy-induced intestinal and cardiac toxicity: current perspectives. Eur J Pharmacol 1007:178282. 10.1016/j.ejphar.2025.178282 41138833

[CR2] Bar-On P, Crews L, Koob AO, Mizuno H, Adame A, Spencer B, Masliah E (2008) Statins reduce neuronal alpha-synuclein aggregation in in vitro models of Parkinson’s disease. J Neurochem 105(5):1656–1667. 10.1111/j.1471-4159.2008.05254.x PMC2822545 18248604

[CR3] Bellini G, D’Antongiovanni V, Palermo G, Antonioli L, Fornai M, Ceravolo R, Bernardini N, Derkinderen P, Pellegrini C (2025) α-Synuclein in Parkinson’s disease: from bench to bedside. Med Res Rev 45(3):909–946. 10.1002/med.22091 PMC11976381 39704040

[CR4] Calabresi P, Di Lazzaro G, Marino G, Campanelli F, Ghiglieri V (2023) Advances in understanding the function of alpha-synuclein: implications for Parkinson’s disease. Brain 146(9):3587–3597. 10.1093/brain/awad150 PMC10473562 37183455

[CR5] Cuervo AM, Stefanis L, Fredenburg R, Lansbury PT, Sulzer D (2004) Impaired degradation of mutant alpha-synuclein by chaperone-mediated autophagy. Science 305(5688):1292–1295. 10.1126/science.1101738 15333840

[CR6] Du RW, Bu WG (2021) Simvastatin Prevents Neurodegeneration in the MPTP Mouse Model of Parkinson’s Disease via Inhibition of A1 Reactive Astrocytes. Neuroimmunomodulation 28(2):82–89. 10.1159/000513678 33735898

[CR7] Ghosh A, Roy A, Matras J, Brahmachari S, Gendelman HE, Pahan K (2009) Simvastatin inhibits the activation of p21ras and prevents the loss of dopaminergic neurons in a mouse model of Parkinson’s disease. J Neurosci 29(43):13543–13556. 10.1523/jneurosci.4144-09.2009 PMC2862566 19864567

[CR8] Huang Q, Chen C, Chen W, Cai C, Xing H, Li J, Li M, Ma S (2023) Cell type- and region-specific translatomes in an MPTP mouse model of Parkinson’s disease. Neurobiol Dis 180:106105. 10.1016/j.nbd.2023.106105 36977454

[CR9] Jeong SH, Lee HS, Chung SJ, Yoo HS, Jung JH, Baik K, Lee YH, Sohn YH, Lee PH (2021) Effects of statins on dopamine loss and prognosis in Parkinson’s disease. Brain 144(10):3191–3200. 10.1093/brain/awab292 34347020

[CR10] Jeong SH, Lee PH (2025) Drug Repositioning and Repurposing for Disease-Modifying Effects in Parkinson’s Disease. J Mov Disord 18(2):113–126. 10.14802/jmd.25008 PMC12061612 39914809

[CR11] Karikari AA, McFleder RL, Ribechini E, Blum R, Bruttel V, Knorr S, Gehmeyr M, Volkmann J, Brotchie JM, Ahsan F, Haack B, Monoranu CM, Keber U, Yeghiazaryan R, Pagenstecher A, Heckel T, Bischler T, Wischhusen J, Koprich JB, Lutz MB, Ip CW (2022) Neurodegeneration by α-synuclein-specific T cells in AAV-A53T-α-synuclein Parkinson’s disease mice. Brain Behav Immun 101:194–210. 10.1016/j.bbi.2022.01.007 35032575

[CR12] Kim MS, Ra EA, Kweon SH, Seo BA, Ko HS, Oh Y, Lee G (2023) Advanced human iPSC-based preclinical model for Parkinson’s disease with optogenetic alpha-synuclein aggregation. Cell Stem Cell 30(7):973–986e911. 10.1016/j.stem.2023.05.015 PMC10829432 37339636

[CR13] Kirik D, Rosenblad C, Burger C, Lundberg C, Johansen TE, Muzyczka N, Mandel RJ, Björklund A (2002) Parkinson-like neurodegeneration induced by targeted overexpression of alpha-synuclein in the nigrostriatal system. J Neurosci 22(7):2780–2791. 10.1523/jneurosci.22-07-02780.2002 PMC6758323 11923443

[CR14] Kirk RGW (2018) Recovering The Principles of Humane Experimental Technique: The 3Rs and the Human Essence of Animal Research. Sci Technol Hum Values 43(4):622–648. 10.1177/0162243917726579 PMC6027778 30008492

[CR15] Koob AO, Ubhi K, Paulsson JF, Kelly J, Rockenstein E, Mante M, Adame A, Masliah E (2010) Lovastatin ameliorates alpha-synuclein accumulation and oxidation in transgenic mouse models of alpha-synucleinopathies. Exp Neurol 221(2):267–274. 10.1016/j.expneurol.2009.11.015 PMC2812599 19944097

[CR16] Kumar A, Sharma N, Gupta A, Kalonia H, Mishra J (2012) Neuroprotective potential of atorvastatin and simvastatin (HMG-CoA reductase inhibitors) against 6-hydroxydopamine (6-OHDA) induced Parkinson-like symptoms. Brain Res 1471:13–22. 10.1016/j.brainres.2012.06.050 22789904

[CR17] Lodge A, Agin-Liebes J (2026) α-Synuclein biomarkers for Parkinson’s disease. Cold Spring Harb Perspect Med. 10.1101/cshperspect.a041944 PMC12863175 40983493

[CR18] Mady A, Nabil Y, Daoud A, Alnajjar AZ, Alsalloum T, Marwan M, Abdelmeseh M, Elsayed M, Krayim A, Alaa M, Masoud M, Bushara N, Faisal R, Ahmed H, Saad M, Belabaci Z, Barrett MJ, Berman B, Negida A (2024) Determining the role of statins in Parkinson’s disease risk reduction and disease modification: a comprehensive meta-analysis of 4 million participants’ data. CNS Neurosci Ther 30(8):e14888. 10.1111/cns.14888 PMC11298167 39097909

[CR19] Marques NF, Castro AA, Mancini G, Rocha FL, Santos ARS, Prediger RD, De Bem AF, Tasca CI (2018) Atorvastatin prevents early oxidative events and modulates inflammatory mediators in the striatum following intranasal 1-methyl-4-phenyl-1,2,3,6-tetrahydropyridine (MPTP) administration in rats. Neurotox Res 33(3):549–559. 10.1007/s12640-017-9840-8 29164519

[CR20] Mazzulli JR, Xu YH, Sun Y, Knight AL, McLean PJ, Caldwell GA, Sidransky E, Grabowski GA, Krainc D (2011) Gaucher disease glucocerebrosidase and α-synuclein form a bidirectional pathogenic loop in synucleinopathies. Cell 146(1):37–52. 10.1016/j.cell.2011.06.001 PMC3132082 21700325

[CR21] Mesentier-Louro LA, Goldman C, Ndayisaba A, Buonfiglioli A, Rooklin RB, Schuldt BR, Uchitelev A, Khurana V, Blanchard JW (2025) Cholesterol-mediated lysosomal dysfunction in APOE4 astrocytes promotes α-synuclein pathology in human brain tissue. bioRxiv. 10.1101/2025.02.09.637107

[CR22] Mohammadi R, Shirazi M, Sadat-Madani SF, Yeo Cheng Long MZ, Singh CL, Tan JY, Deng X, Hashemi Fard SM, Ng SYE, Ng ASL, Tan LCS, Saffari SE (2025) Common SNCA genetic variants and Parkinson’s disease risk: a systematic review and meta-analysis. Int J Mol Sci. 10.3390/ijms26136001 PMC12250285 40649779

[CR23] Morel J, Hargreaves I, Brealey D, Neergheen V, Backman JT, Lindig S, Bläss M, Bauer M, McAuley DF, Singer M (2017) Simvastatin pre-treatment improves survival and mitochondrial function in a 3-day fluid-resuscitated rat model of sepsis. Clin Sci (Lond) 131(8):747–758. 10.1042/cs20160802 28202686

[CR24] Oliveira LM, Falomir-Lockhart LJ, Botelho MG, Lin KH, Wales P, Koch JC, Gerhardt E, Taschenberger H, Outeiro TF, Lingor P, Schüle B, Arndt-Jovin DJ, Jovin TM (2015) Elevated α-synuclein caused by SNCA gene triplication impairs neuronal differentiation and maturation in Parkinson’s patient-derived induced pluripotent stem cells. Cell Death Dis 6(11):e1994. 10.1038/cddis.2015.318 PMC4670926 26610207

[CR25] Prahl JD, Pierce SE, van der Schans EJC, Coetzee GA, Tyson T (2023) The Parkinson’s disease variant rs356182 regulates neuronal differentiation independently from alpha-synuclein. Hum Mol Genet 32(1):1–14. 10.1093/hmg/ddac161 PMC9837835 35866299

[CR26] Ramos Dos Santos L, Duplan E, Debord J, Fremont G, Minniti J, Lauritzen I, Mutez E, Leghay C, Chartier-Harlin MC, Checler F, Alves da Costa C (2026) PRKN/parkin-mediated control of SNCA (synuclein alpha) and chaperone-mediated autophagy are defective in cellular, mice models and Parkinson disease-affected brains. Autophagy. 10.1080/15548627.2026.2669458 PMC13502014 42083315

[CR27] Saeedi Saravi SS, Saeedi Saravi SS, Khoshbin K, Dehpour AR (2017) Current insights into pathogenesis of Parkinson’s disease: Approach to mevalonate pathway and protective role of statins. Biomed Pharmacother 90:724–730. 10.1016/j.biopha.2017.04.038 28419968

[CR28] Shi Z, Han S (2025) Personalized statin therapy: targeting metabolic processes to modulate the therapeutic and adverse effects of statins. Heliyon 11(1):e41629. 10.1016/j.heliyon.2025.e41629 PMC11761934 39866414

[CR29] Shwab EK, Gingerich DC, Man Z, Gamache J, Garrett ME, Crawford GE, Ashley-Koch AE, Serrano GE, Beach TG, Lutz MW, Chiba-Falek O (2024) Single-nucleus multi-omics of Parkinson’s disease reveals a glutamatergic neuronal subtype susceptible to gene dysregulation via alteration of transcriptional networks. Acta Neuropathol Commun 12(1):111. 10.1186/s40478-024-01803-1 PMC11218415 38956662

[CR30] Sneddon LU, Halsey LG, Bury NR (2017) Considering aspects of the 3Rs principles within experimental animal biology. J Exp Biol 220(Pt 17):3007–3016. 10.1242/jeb.147058 28855318

[CR31] Theodore S, Cao S, McLean PJ, Standaert DG (2008) Targeted overexpression of human alpha-synuclein triggers microglial activation and an adaptive immune response in a mouse model of Parkinson disease. J Neuropathol Exp Neurol 67(12):1149–1158. 10.1097/NEN.0b013e31818e5e99 PMC2753200 19018246

[CR32] Verderio P, Lecchi M, Ciniselli CM, Shishmani B, Apolone G, Manenti G (2023) 3Rs principle and legislative decrees to achieve high standard of animal research. Animals Basel. 10.3390/ani13020277 PMC9854901 36670818

[CR33] Volpicelli-Daley LA, Luk KC, Patel TP, Tanik SA, Riddle DM, Stieber A, Meaney DF, Trojanowski JQ, Lee VM (2011) Exogenous α-synuclein fibrils induce Lewy body pathology leading to synaptic dysfunction and neuron death. Neuron 72(1):57–71. 10.1016/j.neuron.2011.08.033 PMC3204802 21982369

[CR34] Yan J, Huang J, Liu A, Wu J, Fan H, Shen M, Lai X, Ma H, Sun W, Yang J, Xu Y (2020a) Atorvastatin improves motor function, anxiety and depression by NOX2-mediated autophagy and oxidative stress in MPTP-lesioned mice. Aging (Albany NY) 13(1):831–845. 10.18632/aging.202189 PMC7835000 33289703

[CR35] Yan J, Liu A, Fan H, Qiao L, Wu J, Shen M, Lai X, Huang J (2020b) Simvastatin improves behavioral disorders and hippocampal inflammatory reaction by NMDA-mediated anti-inflammatory function in MPTP-treated mice. Cell Mol Neurobiol 40(7):1155–1164. 10.1007/s10571-020-00804-7 PMC11448790 32016638

[CR36] Ye H, Robak LA, Yu M, Cykowski M, Shulman JM (2023) Genetics and Pathogenesis of Parkinson’s Syndrome. Annu Rev Pathol 18:95–121. 10.1146/annurev-pathmechdis-031521-034145 PMC10290758 36100231

[CR37] Zhang J, Sun B, Yang J, Chen Z, Li Z, Zhang N, Li H, Shen L (2022) Comparison of the effect of rotenone and 1‑methyl‑4‑phenyl‑1,2,3,6‑tetrahydropyridine on inducing chronic Parkinson’s disease in mouse models. Mol Med Rep. 10.3892/mmr.2022.12607 PMC8809117 35039876

