# Supplementary material for: Atorvastatin Shows Limited Disease-Modifying Effects in an A53T α-Synuclein Mouse Model of Parkinson’s Disease
Source: Neurotox Res. 2026 Sep 9;44(5):51. doi: 10.1007/s12640-026-00825-y (PMC13558405; doi:10.1007/s12640-026-00825-y)
Supplement: Supplementary file 2 [file 12640_2026_825_MOESM1_ESM.doc]

Dear Editor

We are submitting our manuscript entitled "Integrated phenotypic, transcriptomic, and structural profiling reveals the limited impact of atorvastatin in an A53T α-synuclein mouse model of Parkinson's disease" to *Neurotoxicity Research* for consideration of publication.

Prior to formal review, we wish to provide a transparent explanation regarding the presentation of the Western blot images in **Fig. 2** of the manuscript, to ensure full clarity during the evaluation process.

**1. Experimental Design**

This study included three experimental groups:

Wild-type control group (control)

A53T model group (A53T)

Atorvastatin-treated group (A53T+ATO)

Each group comprised six biological replicates, all of which were independently subjected to Western blot analysis. Quantitative data and statistical analyses presented in the bar graphs were derived from all six replicates (n = 6) per group.

**2. Western Blot Image Presentation and Cropping**

During Western blotting, a 15-well precast gel was used. In the loading process, the control group samples were inadvertently loaded twice as an internal reference, resulting in the following original lane order on the membrane:

control-1, control-2, control-3, A53T-1, A53T-2, A53T-3, control-1, control-2, control-3 (duplicate), A53T+ATO-1, A53T+ATO-2, A53T+ATO-3

When preparing the figure, the following adjustments were made:

Cropping of redundant lanes: The duplicate control lanes (lanes 7, 8, and 9) were removed.

Selection of representative lanes: The final displayed lanes are control-1, control-2, control-3, A53T-1, A53T-2, A53T-3, A53T+ATO-1, A53T+ATO-2, A53T+ATO-3, showing three representative samples per group.

It is important to note that the representative blot images display three samples per group, while the quantitative analyses were performed using all six biological replicates (n = 6) . The displayed bands are fully consistent with the quantified data and were not selectively chosen to favor any experimental outcome.

**3. Data Integrity Statement**

We hereby declare that:

The only manipulation performed was the cropping of duplicate control lanes; no bands were cloned, copied, altered in background, or signal-enhanced in any way.

All bands presented in the figure originate from a single membrane and a single exposure, and the original data are authentic and complete.

The three representative bands shown correspond directly to three of the six replicates used in the statistical analysis, ensuring that the quantification accurately reflects the inter-group differences.

**4. Availability of Raw Data**

To support this explanation, we are willing to provide, upon request during the review process, the original full-membrane exposure image of the Western blot described above (i.e., the raw TIFF file containing all lanes: control-1, control-2, control-3, A53T-1, A53T-2, A53T-3, control-1, control-2, control-3, A53T+ATO-1, A53T+ATO-2, A53T+ATO-3). This raw image will verify the loading order, the cropping rationale, and the authenticity of the presented bands.

We fully understand and respect the stringent policies of *Neurotoxicity Research* regarding image integrity and are committed to transparent and ethical scientific publication practices. This proactive disclosure is intended to preclude any potential misinterpretation and to allow reviewers to focus on the scientific merits of our study.

We believe that our manuscript falls within the scope of *Neurotoxicity Research* and provides novel insights into the role of atorvastatin in an A53T α-synuclein model of Parkinson's disease. All authors have reviewed and approved the submission, and we declare no competing interests.

Thank you for your time and consideration. Should you have any questions or require additional materials, please do not hesitate to contact us.

Yours sincerely,

**[Corresponding Author's Name]** MingShu Mo

**[Position]** Director of the Department of Neurology

**[Affiliation]**Department of Neurology, The First Affiliated Hospital of Guangzhou Medical University, Guangzhou, Guangdong, China.

**[Email Address]**momingshu123@163.com

**[Phone Number]**15975632425
